# Ultrasonographic analysis of the hyoid bone distance in individuals with neurogenic oropharyngeal dysphagia

**DOI:** 10.1590/2317-1782/20242022074en

**Published:** 2024-05-31

**Authors:** Simone Galli Rocha Bragato, Roberta Gonçalves da Silva, Larissa Cristina Berti

**Affiliations:** 1 Programa de Pós-graduação em Fonoaudiologia, Universidade Estadual Paulista – UNESP - Marília (SP), Brasil.; 2 Laboratório de Pesquisa e Reabilitação em Disfagia, Departamento de Fonoaudiologia, Universidade Estadual Paulista – UNESP - Marília (SP), Brasil.; 3 Laboratório de Análise Articulatória e Acústica, Departamento de Fonoaudiologia, Universidade Estadual Paulista – UNESP - Marília (SP), Brasil.

**Keywords:** Deglutition, Ultrasonography, Hyoid Bone, Deglutition Disorders, Ultrasonics

## Abstract

To compare the ultrasound measurement of distance from the approximation of the hyoid bone during of the maximum deglutition peak between healthy individuals and neurogenic dysphagic individuals and to verify the effect of food consistencies on the displacement of the hyoid bone. Prospective, controlled clinical study. Ultrasound recordings of the oropharyngeal deglutition were conducted in 10 adults diagnosed with oropharyngeal dysphagia and in 10 healthy adults, matched by sex and age group. A portable ultrasound model Micro ultrasound system with a microconvex transducer 5-10 MHz, coupled to a computer as well as the head stabilizer were used. The ultrasound images were recorded using the AAA software (Articulate Assistant Advanced) at a rate of 120 frames/second. Food consistencies level 0 (free volume and 5 mL) and level 4 (5 mL) were used, based on the International Dysphagia Diet Standardisation Initiative (IDSSI). The calculation of the mean and standard deviation was used for the descriptive analysis, while the repeated measures ANOVA test was used for the inferential analysis. Results showed dysphagic individuals had lower elevation of the hyoid bone marked by a longer distance from the approximation of the hyoid bone during of the maximum deglutition peak when compared to healthy individuals, regardless of the food consistency offered. It was concluded that the ultrasound measurement of distance from the approximation of the hyoid bone during of the maximum deglutition peak showed less laryngeal elevation in individuals with neurogenic oropharyngeal dysphagia when compared to healthy individuals for all food consistencies offered.

## INTRODUCTION

Among the imaging exams used for the quantitative analysis of oropharyngeal swallowing, videofluoroscopic and videoendoscopic assessments of deglutition are commonly employed. However, supplementary methods like ultrasound stand out for their practicality, portability, non-invasiveness, cost-effectiveness, absence of radiation, and the ability to use typical foods during assessments^(1)^. Ultrasonographic analysis has shown its value as an adjuvant tool for studying deglutition biomechanics, as evidenced in previous studies^(1-19)^.

The movement of the hyoid bone is a crucial parameter for analyzing deglutition biomechanics. Its displacement is intimately linked to the timing of the pharyngeal response initiation and the individual’s capability to protect the lower airways while transporting the food bolus to the esophagus^(20)^. This movement signals the larynx’s elevation during oropharyngeal deglutition^(21)^ and can be easily observed through ultrasonography^(19)^. Ultrasound enables the clear observation of hyoid bone movement during the swallowing of food or saliva by researchers and health professionals^(14)^.

The literature suggests that ultrasound can serve as a screening tool for deglutition function, allowing for the accurate determination of both the duration of deglutition and the displacement or trajectory of the hyoid bone movement^(15)^. Studies have indicated that ultrasound helps distinguish between normal and abnormal hyoid bone movements, offering a consistent and complementary measurement for diagnosing deglutition disorders^(3)^.

Investigations into the quantification of hyoid bone displacement using ultrasound have shown high inter- and intra-rater reliability correlation coefficients^(22)^, attesting to its reliability. Another study comparing ultrasound’s accuracy in assessing hyoid bone displacement during deglutition using the gold standard—videofluoroscopy—revealed good precision and high evaluator reliability^(23)^.

The elevation of the larynx and hyoid bone, crucial for initiating the pharyngeal phase of deglutition, is influenced by the physical properties of the ingested food, such as consistency, volume, flavor, and temperature. These factors can affect the hyoid bone’s trajectory during deglutition^(6)^. This elevation is also dependent on the food bolus’s organoleptic characteristics.

Moreover, the oral phase events, particularly the concurrent movements of the jaw, tongue, and food bolus propulsion, are primary regulators of the hyoid bone’s upward displacement during deglutition^(19)^.

Ultrasonographic examination of hyoid bone displacement provides insights into the timing, quality of trajectory, and distance relative to other structures involved in deglutition biomechanics. However, the potential of measuring hyoid bone displacement to augment the analysis of deglutition biomechanics across different food consistencies and volumes in varied populations remains underexplored.

Given the potential differences in deglutition biomechanics between healthy individuals and those with neurogenic oropharyngeal dysphagia, it is anticipated that the latter group will exhibit greater distances in hyoid bone approximation. Additionally, this study aims to explore how food consistencies affect the displacement of the hyoid bone.

Thus, this study aims to compare the ultrasonographic measurement of the hyoid bone’s approximation distance at the peak of deglutition between healthy individuals and those with neurogenic oropharyngeal dysphagia and to assess the impact of food consistencies on the hyoid bone’s displacement.

## METHOD

### Ethical aspects

Case study approved by the Research Ethics Committee of the aforementioned Institution under protocol no. 19946119.8.0000.5406. All participants signed an Informed Consent Form (ICF) prior to study commencement.

### Sample

The study sample comprised 20 adult individuals divided into two groups: the experimental group (EG) consisted of 10 adults with neurogenic oropharyngeal dysphagia of various etiologies (Table 1), whereas the control group (CG) was composed of 10 healthy adults matched by sex and age (4 men and 6 women; average age = 46.9 years).

**Table 1:** Profile of the sample of patients with neurogenic oropharyngeal dysphagia

**Individual**	**Age**	**Sex**	**Underlying Disease**
Dysphagic 1	30	Female	Spinocerebellar Atrophy
Dysphagic 2	32	Male	Cerebral Aneurysm
Dysphagic 3	40	Female	Myasthenia Gravis
Dysphagic 4	42	Male	Guillain-Barré Syndrome
Dysphagic 5	49	Female	Cerebellar Atrophy
Dysphagic 6	53	Female	Cerebellar Atrophy
Dysphagic 7	53	Male	Multiple Sclerosis
Dysphagic 8	55	Male	Stroke
Dysphagic 9	57	Female	Multiple Sclerosis
Dysphagic 10	58	Female	Cerebral Microinfarcts

Convenience sampling was applied to select the EG individuals: aged ≥18 years, of both sexes, able to remain seated, with underlying neurological pathologies diagnosed by a medical team, and with oropharyngeal dysphagia diagnosed by clinical evaluation and videoendoscopic assessment of deglutition.

To recruit the CG individuals, a checklist (Chart 1) was applied to screen for factors that might suggest any functional difficulties and/or changes in deglutition. The individual was excluded from the study sample if any item on the checklist was checked.

**Chart 1:** Checklist for screening swallowing difficulties in healthy individuals

**Questions**	**YES**	**NO**
Use of antidepressant medication?		
Dental prosthesis?		
Cancer in the head or neck region?		
Neurological and neurodegenerative diseases?		
Difficulty in feeding and/or swallowing?		

### Equipment

Data were collected using a portable Micro-Ultrasound System equipped with a microconvex transducer (5-10 MHz, 10 mm radius, 150 degree max. field of view), coupled to a computer, and a head stabilizer. Ultrasound images for each swallowing were recorded on Articulate Assistant Advanced (AAA) software at a rate of 120 frames per second.

### Experimental procedure

Ultrasonographic assessment of deglutition was conducted with participants sitting in a comfortable position. The head stabilizer was adjusted for each individual, to couple the microconvex transducer at 90 degrees to the submandibular region. Conductive gel was applied for impedance coupling between the transducer surface and the individual’s submandibular region skin, facilitating the formation of the image of the tongue surface and the hyoid bone in the sagittal plane.

After explaining the ultrasonographic examination to the individuals, they were instructed to swallow the food that would be offered to them in disposable spoons and cups.

Food consistencies of level 0 (5 mL and free volume)—which consisted of free swallowing, that is, without specific volume measurement made by the patient—and level 4 (5 mL) were used following the International Dysphagia Diet Standardization Initiative (IDSSI) guidelines^(24)^. Filtered water was used for the level 0 consistency (thin liquid), while filtered water, food thickener, and diet peach-flavored juice were utilized in the preparation of level 4 consistency (extremely thickened liquid). The foods were offered in the following order: level 4 consistency (5 mL), level 0 consistency (5 mL), and level 0 consistency (free volume). For each situation, two offerings were made, generating 120 ultrasonographic swallowing videos (20 individuals analyzed under three food consistency conditions and volumes for two offerings).

### Ultrasonographic analysis of the hyoid bone

**Figure 1:**
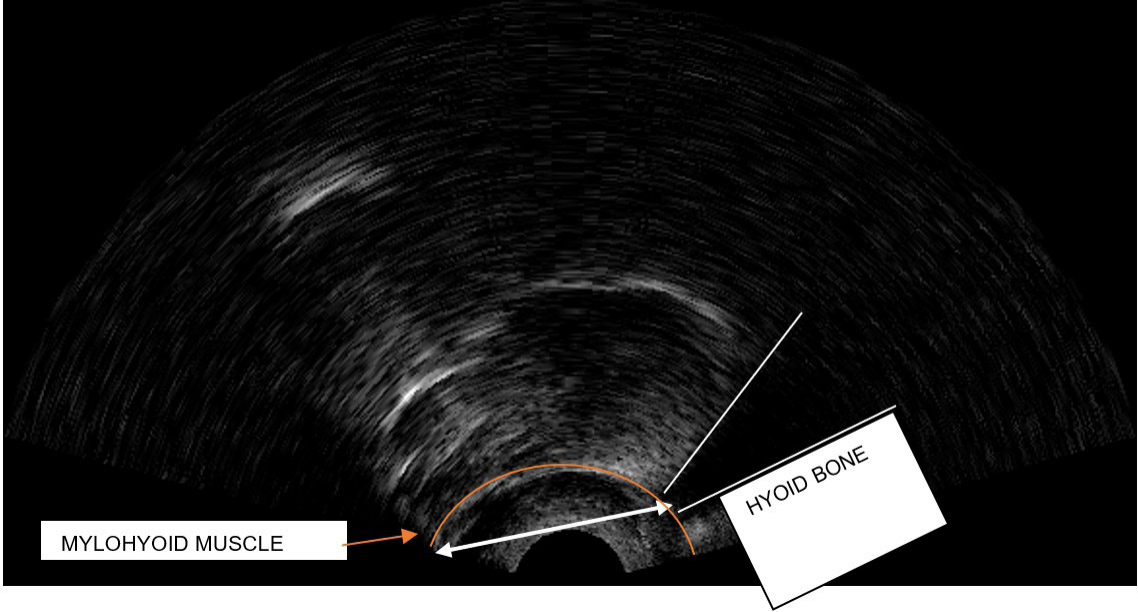
Arrow indicating the hyoid bone’s approximation distance at the peak of deglutition in a dysphagic individual

**Figure 2:**
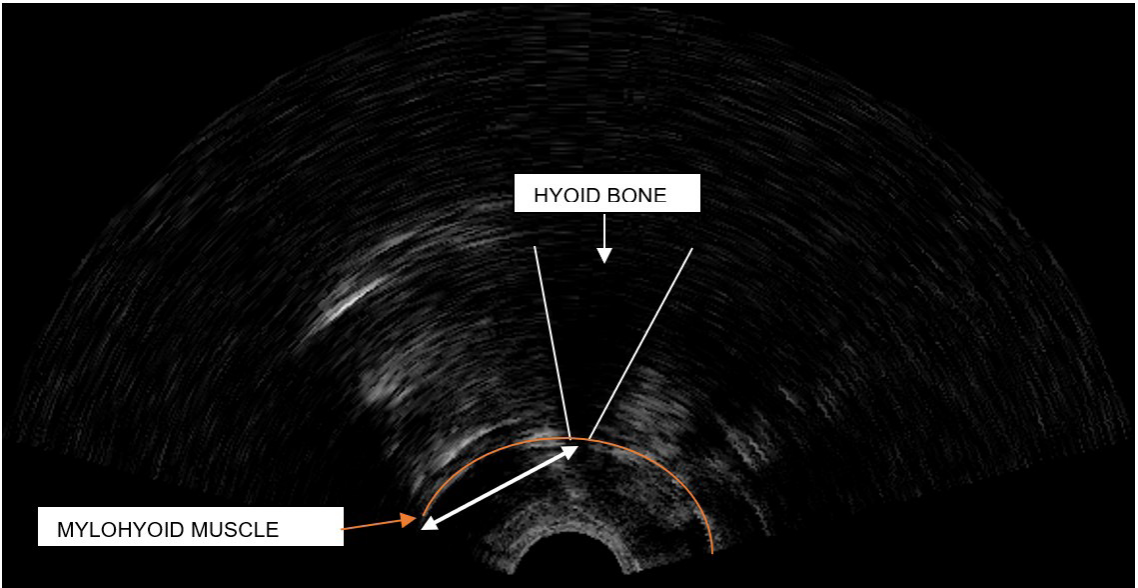
Arrow indicating the hyoid bone’s approximation distance at the peak of deglutition in a healthy individual

The parameter used for quantitative analysis was the hyoid bone’s approximation distance at the peak of deglutition, thus the frame corresponding to the maximum approximation of the hyoid bone to the mylohyoid muscle was selected, and the distance between the bottom part of the hyoid bone and the insertion of the mylohyoid muscle was measured, as illustrated in Figures 1 and 2. The measures were taken in centimeters.

Both figures show two arrows each, which indicate the hyoid bone’s approximation distance at the peak of deglutition. Analysis of these figures evidence that the lower the hyoid bone’s elevation, the greater its distance (Figure 1); conversely, the higher the hyoid bone’s elevation, the lesser its distance (Figure 2).

Analysis was also performed using the AAA software. The frame corresponding to the maximum displacement of the hyoid bone was selected to measure the distance. The distance between the bottom part of the hyoid bone and the insertion of the mylohyoid muscle was selected, and the software automatically measured it. This selection was made by the first author of this study, who has previous experience in ultrasonographic analysis. The examiner also re-analyzed 20% of the data within a 90-day interval. The interrater reliability index, that is, the intraclass correlation coefficient (ICC), was 0.80, demonstrating the excellent reliability of the results.

### Statistical analysis

Descriptive and inferential statistical treatments of the data were performed using the Statistica 7.0 software. The descriptive analysis employed mean and standard deviation calculations for the hyoid bone’s approximation distance at the peak of deglutition, whereas the inferential analysis utilized the repeated measures ANOVA test to compare the measurements of the hyoid bone’s approximation distance considering the groups and the food consistencies. A significance level of 5% (p≤0.05) was adopted for all statistical analyses.

## RESULTS

Table 2 presents the mean and standard deviation of the hyoid bone’s approximation distance at the peak of deglutition according to groups and food consistencies.

**Table 2:** Mean and standard deviation values of the hyoid bone’s approximation distance (in centimeters) at the peak of deglutition in dysphagic and healthy individuals by food consistency and volume

**Food Consistency**	**Level 0 - Free Volume**	**Level 0 - 5ml**	**Level 4 - 5ml**
**Group**	**Mean (SD)**	**Mean (SD)**	**Mean (SD)**
EG	3.37 (±0.50)	3.31 (±0.62)	3.22 (±0.43)
CG	2.86 (±0.42)	2.82 (±0.36)	2.77 (±0.33)

**Caption:** SD: Standard Deviation; EG: Experimental Group (dysphagic individuals); GC: Control Group (healthy individuals)

Table 2 shows that, regardless of the food consistencies (level 0 - free volume, level 0 - 5 mL, and level 4 - 5 mL), the values for the EG are numerically higher than those for the CG.

**Figure 3:**
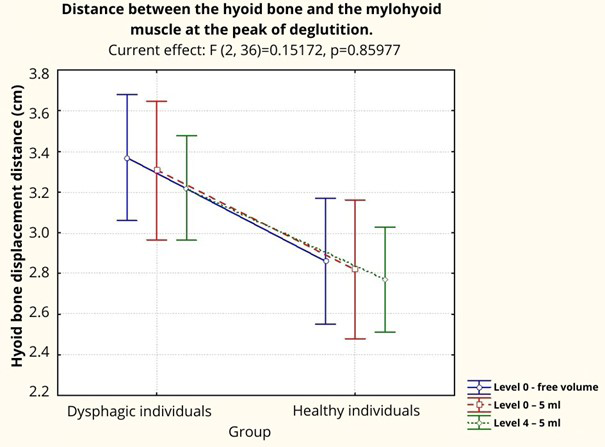
Comparison of the hyoid bone’s approximation distance at the peak of deglutition between dysphagic and healthy individuals

In the repeated measures ANOVA statistical test, a significant effect was observed only regarding the groups (*F*(1, 18) = 6.08, *p*<0.05). No significant effects were observed concerning the food consistencies (*F*(2, 36) = 2.45, *p*>0.05) or for the interaction between group and food consistency (*F*(2, 36) = 0.15, *p*>0.05). The EG showed a greater hyoid bone approximation distance at the peak of deglutition compared to the CG regardless of the offered food consistency (Figure 3).

## DISCUSSION

This study aimed to measure the displacement of the hyoid bone through ultrasonographic evaluation and compare it between healthy adults and those with neurogenic oropharyngeal dysphagia, as well as examine the effect of food consistency in both groups.

The results indicated that there was reduced elevation of the hyoid bone during swallowing in the EG across all food consistencies and volumes offered, meaning there was a greater hyoid bone approximation distance compared with that of the CG. In the literature, various studies using ultrasound have also reported this finding^(8,11,25-27)^; however, these studies did not use varied food consistencies and volumes in their analysis. One study, in particular, expanded its conclusion by suggesting that ultrasonographic evaluation could be used as a screening tool for dysphagic patients, indicating that hyoid bone displacement values <13.5 mm should be used as a cutoff point to detect laryngotracheal penetration or aspiration (26). Nevertheless, the suggestion of using this ultrasonographic finding as a risk marker for aspiration requires further studies on its accuracy.

It is worth noting that although the aforementioned studies adopted different reference points to calculate the hyoid bone’s displacement, such as the distance between the hyoid bone and the mandible^(26,27)^ or that between the hyoid bone and the thyroid cartilage^(8,11,25)^, all of them reported a difference in hyoid bone displacement values between dysphagic and healthy individuals, confirming the potential of this examination to assist in the qualitative and quantitative investigation of this swallowing parameter. However, it should be emphasized that such measurements have not been explored across different food consistencies and volumes so far.

Such difference in ultrasonographic measurements found between the groups is due to the excursion of the larynx/hyoid bone, which occurs completely in healthy individuals, as there is no underlying disease affecting its movement. In contrast, in dysphagic individuals, in general, because of the neurogenic disease, the corticobulbar fibers, the structures of the nucleus of the solitary tract, and the nucleus ambiguus induce both the representation of the modulation of the pharyngeal response and the weakness of the pharynx, laryngeal muscles, and the muscles innervating the hyoid bone, resulting in reduced laryngeal elevation and decreased hyoid-laryngeal approximation^(11,25)^.

Only two studies^(28,29)^ did not find a statistical difference in the displacement of the hyoid bone in patients with dysphagia when compared to healthy individuals. The first study^(28)^ explains this fact from the underlying disease – Parkinson’s Disease (PD); dysphagia in patients with PD is associated with dysfunction in oropharyngeal swallowing, specifically in lingual bradykinesia, hesitation movement, and tongue tremor. In patients with PD, there is a significantly delayed interval between the onset of tongue movement and the shorter hyoid-thyroid approximation distance, supporting the premise that the dyskinesia occurring in the oral phase does not result in the displacement of the hyoid bone itself when assessed by ultrasound in this population. Meanwhile, the second study^(29)^ reports that a compensatory strategy for hyoid bone elevation was employed in individuals with dysphagia. While individuals in the control group consumed the liquid consistency (water), those in the experimental group ingested water with viscosity adjustments depending on the degree of dysphagia impairment, and thus the same hyoid bone displacement during deglutition was observed in both groups.

Previous studies^(11,25-27)^ have also been emphatic in highlighting the accuracy of the ultrasound examination in assessing the elevation of the hyoid bone compared with the videofluoroscopic assessment of deglutition, indicating that ultrasound showed high sensitivity and specificity with the videofluoroscopy measures of hyoid bone displacement.

Regarding the effect of food consistencies, statistical difference was found between the different food consistencies offered to the participants. Although a numerical difference showing a tendency for that was observed, it was not statistically significant.

The literature shows that most ultrasonographic studies that analyzed the displacement of the hyoid bone during swallowing in patients with dysphagia and compared it with that of healthy individuals did so using a single consistency – liquid^(8,11,25,26,28)^, or during saliva swallowing^(27)^. Of these, only a recent study (30) concluded that the displacements of the hyoid bone and the larynx were not affected by food viscosity, as the change in food consistency did not significantly alter the contraction rate of the laryngeal muscle.

This study presents some limitations that should be considered in analyzing the evidence found. The small sample size, the heterogeneity of dysphagic patients – both in terms of disease etiology and of age and dysphagia severity, as well as the participants’ anthropometric variables, should prompt reflection on the individual differences in hyoid bone elevation and also in the performance of the pharyngeal response provoked in each of the neurogenic etiologies. Conversely, the findings suggest that this ultrasonographic measurement in varied food consistencies can be used complementarily to the evaluation of oropharyngeal dysphagia. These results will provide clinicians with a parameter of laryngeal elevation in this population, through a low-cost, non-invasive technique and a safe and comfortable procedure using portable equipment.

## CONCLUSION

The ultrasonographic measurement of the hyoid bone’s approximation distance at the peak of deglutition indicated reduced laryngeal elevation in individuals with neurogenic oropharyngeal dysphagia compared to healthy individuals across all tested food consistencies.
