# Supplementary material for: Ultrasonographic analysis of the hyoid bone distance in individuals with neurogenic oropharyngeal dysphagia
Source: Codas. 2024 May 31;36(3):e20220074. doi: 10.1590/2317-1782/20242022074en (PMC11166034; doi:10.1590/2317-1782/20242022074en)
Supplement: Translation

# Translation: Análise ultrassonográfica da distância do osso hioide em indivíduos com disfagia orofaríngea neurogênica

**DOI:** 10.1590/2317-1782/20242022074pt

**Authors:** Simone Galli Rocha Bragato, Roberta Gonçalves da Silva, Larissa Cristina Berti

**Affiliations:** 1 Programa de Pós-graduação em Fonoaudiologia, Universidade Estadual Paulista – UNESP - Marília (SP), Brasil.; 2 Laboratório de Pesquisa e Reabilitação em Disfagia, Departamento de Fonoaudiologia, Universidade Estadual Paulista – UNESP - Marília (SP), Brasil.; 3 Laboratório de Análise Articulatória e Acústica, Departamento de Fonoaudiologia, Universidade Estadual Paulista – UNESP - Marília (SP), Brasil.

**Keywords:** Deglutição, Ultrassonografia, Osso Hióide, Transtornos de Deglutição, Ultrassom

## Abstract

Comparar a medida ultrassonográfica de distância da aproximação do osso hioide no momento do pico máximo da deglutição entre indivíduos saudáveis e disfágicos neurogênicos e verificar o efeito das consistências do alimento no deslocamento do osso hioide. Estudo clínico prospectivo controlado. Foram realizadas avaliações ultrassonográficas da deglutição orofaríngea em 10 adultos com diagnóstico de disfagia orofaríngea e 10 adultos saudáveis, pareados por sexo e faixa etária. Para tanto, foi utilizado ultrassom portátil com transdutor microconvex 5-10 MHz, além de estabilizador de cabeça. As imagens ultrassonográficas foram gravadas pelo software Articulate Assistant Advanced a uma taxa de 120 quadros/segundo. Foram utilizadas as consistências de alimentos nível 0 (volume livre e 5 mL) e nível 4 (5 mL), conforme as recomendações do International Dysphagia Diet Standardisation Initiative. A distância foi mensurada no momento do pico máximo da deglutição entre a parte inferior do osso hioide e a inserção do músculo milo-hioideo. Cálculos de média e o desvio padrão foram utilizados na análise descritiva, enquanto o teste ANOVA de medidas repetidas foi aplicado na análise inferencial.Resultados evidenciaram que indivíduos disfágicos apresentaram menor elevação do osso hioide, marcada por maior distância da aproximação do osso hioide no momento do pico máximo da deglutição quando comparados aos indivíduos saudáveis, independentemente da consistência alimentar ofertada. Concluiu-se que a medida ultrassonográfica de distância da aproximação do osso hioide no momento do pico máximo da deglutição mostrou menor elevação laríngea em indivíduos com disfagia orofaríngea neurogênica quando comparados aos indivíduos saudáveis para todas as consistências alimentares ofertadas.

## INTRODUÇÃO

Dentre os exames de imagem com análise quantitativa utilizados para avaliar a deglutição orofaríngea, a videofluoroscopia e a videoendoscopia da deglutição são as comumente usadas. Entretanto, existem métodos complementares para auxiliar no diagnóstico e na caracterização de alguns aspectos da deglutição orofaríngea. Dentre eles, o ultrassom tem sido evidenciado por sua praticidade, portabilidade, por ser uma ferramenta não invasiva, de baixo custo, livre de radiação e com possibilidade de utilizar alimentos usuais nas avaliações^(1)^. As contribuições da análise ultrassonográfica podem ser utilizadas como achados complementares para análise da biomecânica da deglutição como foi observado em estudos prévios^(1-19)^.

O deslocamento do osso hioide é um parâmetro importante na avaliação da biomecânica da deglutição, uma vez que sua excursão está intimamente relacionada ao tempo de início da resposta faríngea e à capacidade do indivíduo em proteger a via aérea inferior durante o transporte de bolo alimentar para o esôfago^(20)^, na medida em que sinaliza a elevação da laringe durante a deglutição orofaríngea^(21)^. Esse deslocamento pode ser facilmente visualizado no exame ultrassonográfico^(19)^. A ultrassonografia é uma técnica que pesquisadores e profissionais da saúde podem facilmente observar o movimento do osso hioide durante a deglutição de alimentos ou saliva^(14)^.

A literatura também sugere que a ultrassonografia pode ser utilizada como ferramenta para triagem da função da deglutição. Além disso, é possível determinar com precisão tanto a duração da deglutição, quanto o deslocamento ou trajetória do movimento do osso hioide pelo ultrassom^(15)^. Pesquisadores^(3)^ indicaram que a ultrassonografia auxilia na discriminação entre movimentos normais e anormais do osso hioide sendo um método que propicia mensuração altamente consistente e complementar para o diagnóstico dos distúrbios da deglutição.

Quando investigado a viabilidade de quantificar o deslocamento do osso hioide utilizando a ultrassonografia por meio da confiabilidade inter e intra-avaliadores, foi verificado alto coeficiente de correlação^(22)^ entre as medidas extraídas pelos examinadores, indicando sua confiabilidade. Outro estudo recente verificou a acurácia do uso da ultrassonografia para avaliar o deslocamento do osso hioide durante a deglutição, a partir da confiabilidade dos examinadores, comparando sua precisão com o exame padrão ouro da deglutição, ou seja, a videofluoroscopia. Os resultados encontrados mostraram boa precisão e alta confiabilidade entre os avaliadores^(23)^.

Em relação a elevação da laringe e do osso hioide verifica-se que estes mecanismos são acionados na deglutição quando iniciada a fase faríngea por meio do início da resposta faríngea. A literatura tem reportado^(6)^ que as propriedades físicas do alimento ingerido, como a consistência, volume, sabor e temperatura do bolo alimentar, podem influenciar na trajetória do osso hioide durante a deglutição. Isso significa dizer que a elevação do osso hioide tem codependência das características organolépticas do bolo alimentar.

Além das características organolépticas do bolo alimentar, eventos que ocorrem na fase oral da deglutição, em especial os movimentos concomitantes da mandíbula, da língua e da propulsão do bolo alimentar são os principais reguladores do deslocamento ascendente do osso hioide durante a deglutição^(19)^.

A observação do deslocamento do osso hioide pelo exame ultrassonográfico permite extrair medidas quanto ao tempo do deslocamento, qualidade da trajetória do osso hioide, além da distância em relação à outras estruturas envolvidas na biomecânica da deglutição.

Destaca-se, porém, que apesar da viabilidade de se adotar a medida de deslocamento do osso hioide para complementar a análise da biomecânica da deglutição, esse dado não foi explorado em diferentes consistências e volumes de alimentos em populações distintas.

Assim, assumindo que há uma diferença na biomecânica da deglutição entre indivíduos saudáveis e disfágicos, espera-se que indivíduos com disfagia orofaríngea neurogênica apresentem maiores valores de distância da aproximação do osso hioide comparativamente aos sujeitos sem disfagia orofaríngea neurogênica. Além disso, considerando o impacto das consistências alimentares na modulação da fase oral da deglutição e no acionamento da resposta faríngea, espera-se encontrar diferenças no deslocamento do osso hioide em função da consistência alimentar.

Portanto, este estudo tem por objetivo comparar a medida ultrassonográfica de distância da aproximação do osso hioide no momento do pico máximo da deglutição entre indivíduos saudáveis e disfágicos neurogênicos, e verificar o efeito das consistências do alimento no deslocamento do osso hioide.

## MÉTODO

### Aspectos éticos

Estudo de caso aprovado pelo Comitê de Ética em Pesquisa sob o protocolo nº 19946119.8.0000.5406. Todos os indivíduos incluídos na pesquisa tiveram ciência e assinaram o termo de consentimento livre e esclarecido.

### Casuística

Participaram deste estudo 20 indivíduos adultos divididos em dois grupos. O grupo experimental (GE) foi composto por 10 adultos com disfagia orofaríngea neurogênica de distintas etiologias, conforme mostra a Tabela 1 e o grupo controle (GC) constituído por 10 indivíduos adultos saudáveis, pareados por sexo e idade, sendo 4 sujeitos do sexo masculino, 6 sujeitos do sexo feminino com média de idade de 46,9 anos.

**Tabela 1:** Perfil da amostra dos pacientes com disfagia orofaríngea neurogênica

Indivíduos	Idade	Sexo	Doença de base
Disfágico 1	30	Feminino	Atrofia Espinocerebelar
Disfágico 2	32	Masculino	Aneurisma Cerebral
Disfágico 3	40	Feminino	Miastenia Gravis
Disfágico 4	42	Masculino	Guillain-Barré
Disfágico 5	49	Feminino	Atrofia Cerebelar
Disfágico 6	53	Feminino	Atrofia Cerebelar
Disfágico 7	53	Masculino	Esclerose Múltipla
Disfágico 8	55	Masculino	Acidente Vascular Encefálico
Disfágico 9	57	Feminino	Esclerose Múltipla
Disfágico 10	58	Feminino	Microinfartos cerebrais

Os indivíduos do GE foram selecionados por conveniência, com idade igual e/ou superior a 18 anos, ambos os sexos, que conseguissem permanecer na posição sentada e com patologias de base neurológica diagnosticada por equipe médica e com disfagia orofaríngea diagnosticada por avaliação clínica e videoendoscópica da deglutição.

Na seleção dos indivíduos saudáveis foi aplicado um *checklist* (Quadro 1) para o rastreamento de fatores que sugerissem qualquer dificuldade e/ou alteração na função da deglutição. No caso da presença de apenas um item do *checklist*, o indivíduo foi excluído da amostra.

**Quadro 1:** *Check-list* para rastreamento da dificuldade de deglutição no indivíduo denominado saudável

Responda sim ou não para as perguntas abaixo:	**SIM**	**NÃO**
Uso de medicamentos antidepressivos?		
Prótese dentária?		
Câncer em região de cabeça ou pescoço?		
Doenças neurológicas e neurodegenerativas?		
Dificuldade para se alimentar e/ou para deglutir?		

### Equipamento

Para a coleta dos dados foi utilizado ultrassom portátil modelo Micro ultrasound system com transdutor microconvex 5-10 MHz, 10 mm radius, 150 *degree max field of view* acoplado a um computador, além do estabilizador de cabeça. As imagens ultrassonográficas referentes a cada uma das deglutições foram gravadas pelo software AAA (*Articulate Assistant Advanced*) em uma taxa de 120 frames/segundo.

### Procedimento experimental

A avaliação ultrassonográfica da deglutição foi conduzida com os participantes sentados em uma posição confortável. O estabilizador de cabeça foi ajustado em cada um dos indivíduos, de modo a acoplar o transdutor micro-convexo a 90º com a região submandibular. Foi utilizado o gel condutor para acoplamento de impedância entre a superfície do transdutor e a superfície cutânea da região submandibular do indivíduo, propiciando a formação da imagem da superfície da língua e do osso hioide no plano sagital.

Após a explanação do exame ultrassonográfico aos indivíduos, os mesmos foram orientados a deglutir os alimentos que lhe seriam ofertados em colheres e copos descartáveis.

Foram utilizadas as consistências de alimentos nível 0 no volume de 5 mL e volume livre, que consistiu em deglutição livre, ou seja, sem mensuração de volume específico realizada pelo próprio paciente e nível 4 (5 mL) conforme o *International Dysphagia Diet Standardisation Initiative (IDSSI)* ^(24)^ *.* Para a consistência nível 0 (líquido ralo) foi utilizado água filtrada e para a preparação da consistência nível 4 (líquido extremamente espessado) foi utilizado água filtrada, espessante de alimentos e suco dietético sabor pêssego. Os alimentos foram ofertados, iniciando-se pela consistência nível 4 com volume de 5 mL, consistência nível 0 com volume de 5 mL e, posteriormente, com volume livre. Para cada situação foram realizadas duas ofertas, gerando 120 vídeos ultrassonográficos da deglutição (20 indivíduos analisados em três condições de consistências e volumes de alimentos por duas ofertas).

### Análise ultrassonográfica do osso hioide

O parâmetro utilizado para a análise quantitativa foi a distância da aproximação do osso hioide no momento do pico máximo da deglutição, assim selecionou-se o frame correspondente a máxima aproximação do osso hioide ao músculo milo-hióideo e a mensuração da distância entre a parte inferior do osso hioide e a inserção do músculo milo-hióideo foi realizada, conforme ilustra as imagens da Figura 1 e da Figura 2. Medida mensurada em centímetros.

**Figura 1:** Seta indicando a distância da aproximação do osso hioide no momento do pico máximo da deglutição no indivíduo disfágico

**Figura 2:** Seta indicando a distância da aproximação do osso hioide no momento do pico máximo da deglutição no indivíduo saudável

Em ambas as figuras pode-se visualizar duas flechas, as quais indicam a distância da aproximação do osso hioide no momento do pico máximo da deglutição. A análise dessas figuras permite ilustrar que quanto menor a elevação do osso hioide, maior será a sua distância (Figura 1); e ao contrário, quanto maior a elevação do osso hioide, menor será sua distância (Figura 2).

Para análise, a qual também foi realizada no software AAA (*Articulate Assistant Advanced*), selecionou-se o frame correspondente ao máximo deslocamento do osso hioide afim de realizar a medida da distância. A distância entre a parte inferior do osso hioide e a inserção do músculo milo-hióideo foi selecionada e o próprio *software* mensurou automaticamente o valor da medida. Essa seleção foi feita pelo primeiro autor do estudo, com experiência prévia em análise ultrassonográfica. O examinador também reanalisou 20% dos dados em um intervalo de 90 dias. O índice da confiabilidade intra-examinador, ou seja, o coeficiente de correlação intraclasse (ICC) foi de 0,80 evidenciando o resultado como índice de confiabilidade excelente.

### Análise estatística

Foram realizados tratamentos estatísticos descritivo e inferencial dos dados com o uso do *software* Statistica (versão 7.0). Para a análise descritiva da distância da aproximação do osso hioide no momento do pico máximo da deglutição foi utilizado o cálculo da média e o desvio padrão, enquanto para análise inferencial foi utilizado o teste ANOVA de Medidas Repetidas para comparar as medidas da distância da aproximação do osso hioide, considerando os grupos, e as consistências alimentares. Estabeleceu-se um nível de significância p≤0,05.

## RESULTADOS

A Tabela 2 apresenta a média e desvio padrão da distância da aproximação do osso hioide no momento do pico máximo da deglutição de acordo com os grupos e as consistências de alimento.

**Tabela 2:** Valores médios e desvio padrão da distância (em centímetros) da aproximação do osso hioide no momento do pico máximo da deglutição nos indivíduos disfágicos e saudáveis por consistência e volume de alimento

Consistência Alimentar	Nível 0	Nível 0	Nível 4
	Volume Livre	Volume 5ml	Volume 5ml
Grupo	Média (DP)	Média (DP)	Média (DP)
GE	3,37 (±0,50)	3,31 (±0,62)	3,22 (±0,43)
GC	2,86 (±0,42)	2,82 (±0,36)	2,77 (±0,33)

**Legenda:** DP: desvio padrão; GE: grupo experimental (indivíduos disfágicos); GC: grupo controle (indivíduos saudáveis)

Verificou-se na Tabela 2 que independentemente das consistências de alimentos (nível 0 - volume livre, nível 0 - volume de 5 mL e nível 4 - volume de 5 mL) os valores para os indivíduos disfágicos são numericamente maiores do que os valores para os indivíduos saudáveis.

No teste estatístico ANOVA de medidas repetidas constatou-se efeito significante apenas para o grupo (F (1, 18) = 6,08, p<0,05). Não foi observado efeito significante para as consistências do alimento (F (2, 36) = 2,45, p>0,05) e nem para a interação entre grupo e consistência do alimento (F (2, 36) = 0,15, p>0,05). Os indivíduos disfágicos apresentaram maior distância da aproximação do osso hioide no momento do pico máximo da deglutição quando comparados aos indivíduos saudáveis independentemente da consistência alimentar ofertada, conforme ilustra a Figura 3.

**Figura 3:** Comparação da distância da aproximação do osso hioide no momento do pico máximo da deglutição entre indivíduos disfágicos e saudáveis

## DISCUSSÃO

O presente estudo propôs mensurar o deslocamento do osso hioide por meio da avaliação ultrassonográfica, comparando indivíduos adultos saudáveis e com disfagia orofaríngea neurogênica, além de verificar o efeito da consistência alimentar em ambos os grupos.

Os resultados mostraram que houve menor elevação do osso hioide durante a deglutição no grupo de indivíduos com disfagia orofaríngea neurogênica em todas as consistências e volume de alimentos ofertados, ou seja, houve maior distância da aproximação do osso hioide quando comparados aos indivíduos saudáveis.

Na literatura diversos estudos utilizando o ultrassom também concluíram que houve menor deslocamento do osso hioide nos pacientes disfágicos^(8,11,25-27)^ comparativamente à indivíduos saudáveis, entretanto tais estudos não utilizaram diferentes consistências e volumes de alimento em sua análise. Um estudo, em particular, ampliou sua conclusão sugerindo que a avaliação ultrassonográfica possa ser utilizada como uma ferramenta de triagem para pacientes disfágicos, indicando que o deslocamento do osso hioide com valores abaixo de 13,5 mm deve ser utilizado como ponto de corte para detectar penetração ou aspiração laringotraqueal^(26)^. No entanto, a sugestão deste achado ultrassonográfico ser utilizado como marcador de risco para aspiração exige estudos sobre sua acurácia.

Cabe destacar que apesar dos estudos citados acima adotarem diferentes pontos de referência para calcular o deslocamento do osso hioide, como a distância entre o osso hioide e a mandíbula^(26,27)^ ou osso hioide e cartilagem tireoide^(8,11,25)^, todos reportaram a diferença de valores no deslocamento do osso hioide em indivíduos disfágicos e saudáveis confirmando a possibilidade deste exame auxiliar na investigação qualitativa e quantitativa deste parâmetro da deglutição. No entanto, destaca-se que tais mensurações não foram exploradas em diferentes consistências e volumes de alimentos até o presente momento.

Tal diferença nas medidas ultrassonográficas encontradas entre os grupos se dá pela excursão da laringe/osso hioide que desempenham seu papel integral nos indivíduos saudáveis, pois não há nenhuma doença de base que acarrete na sua movimentação, contrariamente aos indivíduos disfágicos que, de modo geral, devido a doença neurogênica, as fibras corticobulbares, as estruturas do núcleo do trato solitário e do núcleo ambíguo induzem tanto a representação da modulação da resposta faríngea quanto a fraqueza da faringe, dos músculos laríngeos e dos músculos que inervam o osso hioide resultando em uma menor elevação laríngea, assim como a diminuição na aproximação hioide-laringe^(11,25)^.

Apenas dois estudos^(28,29)^ não encontraram diferença estatística no deslocamento do osso hioide em pacientes com disfagia quando comparados a indivíduos saudáveis. O primeiro estudo^(28)^ explica tal fato a partir da doença de base, a Doença de Parkinson (DP), a disfagia em pacientes com DP está relacionado à disfunção da deglutição orofaríngea, especificamente na bradicinesia lingual, no movimento de hesitação e tremor da língua. Em pacientes com DP, há um intervalo significativamente atrasado entre o início do movimento da língua e a distância mais curta da aproximação hioide-tireoide, apoiando a premissa de que a discinesia que ocorre na fase oral não acarreta o deslocamento do osso hioide, propriamente dito, quando visualizado pelo US nessa população. Já o segundo estudo^(29)^ relata que foi realizada uma estratégia compensatória para elevação do osso hioide nos indivíduos disfágicos. Enquanto indivíduos do grupo controle ingeriram a consistência líquida (água), os indivíduos do grupo alvo ingeriram água com ajustes de viscosidade dependendo do grau de comprometimento da disfagia, e assim foi verificado o mesmo deslocamento do osso hioide durante a deglutição em ambos os grupos.

As pesquisas^(11,25-27)^ também foram contundentes em destacar a acurácia do exame de ultrassonografia para avaliar a elevação do osso hioide em relação ao exame de videofluoroscopia da deglutição, indicando que o ultrassom apresentou alta sensibilidade e especificidade com as medidas da videofluoroscopia do deslocamento do osso hioide.

Em relação ao efeito das consistências alimentares, verificou-se que não houve diferença estatística nas distintas consistências de alimento ofertada aos participantes. Embora tenha sido possível observar uma diferença numérica mostrando uma tendência para tal diferença, esta não se mostrou estatisticamente significante.

A literatura mostra que grande parte dos estudos ultrassonográficos que analisaram o deslocamento do osso hioide durante a deglutição em pacientes disfágicos e compararam com indivíduos saudáveis o fizeram somente em uma única consistência, a líquida^(8,11,25,26,28)^ ou durante a deglutição de saliva^(27)^. Desses, somente um estudo recente^(29)^ concluiu que o deslocamento do osso hioide e da laringe não foi afetado pela viscosidade do alimento, pois a mudança da consistência alimentar não alterou significativamente a taxa de contração da musculatura laríngea.

Esta pesquisa possui algumas limitações que devem ser consideradas na análise das evidências encontradas. O reduzido tamanho da amostra, a heterogeneidade dos pacientes disfágicos, tanto em termos de etiologia da doença, quanto pela idade e gravidade da disfagia, e também as variáveis antropométricas dos indivíduos devem promover reflexão sobre as diferenças individuais na elevação do osso hioide e também no desempenho da resposta faríngea provocada em cada uma das etiologias neurogênicas. Por outro lado, os achados encontrados sugerem que essa medida ultrassonográfica em diferentes consistências de alimento possa ser utilizada de modo complementar à avaliação da disfagia orofaríngea. Estes resultados permitirão aos clínicos um parâmetro da elevação laríngea nesta população, por meio de um exame de baixo custo, com técnica não invasiva, procedimento seguro e confortável com equipamento portátil.

## CONCLUSÃO

A medida ultrassonográfica de distância da aproximação do osso hioide no momento do pico máximo da deglutição mostrou menor elevação laríngea em indivíduos com disfagia orofaríngea neurogênica, quando comparados aos indivíduos saudáveis em diferentes consistências de alimentos testados.
